# Elevated Trehalose Levels in *C. elegans daf-2* Mutants Increase Stress Resistance, Not Lifespan

**DOI:** 10.3390/metabo11020105

**Published:** 2021-02-12

**Authors:** Madina Rasulova, Aleksandra Zečić, Jose Manuel Monje Moreno, Lieselot Vandemeulebroucke, Ineke Dhondt, Bart P. Braeckman

**Affiliations:** Laboratory of Aging Physiology and Molecular Evolution, Department of Biology, Ghent University, K.L. Ledeganckstraat 35, B-9000 Ghent, Belgium; madina.rasulova@kuleuven.be (M.R.); Aleksandra.Zecic@UGent.be (A.Z.); jmmonmor@upo.es (J.M.M.M.); Lieselot.Vandemeulebroucke@UGent.be (L.V.); ineke.dhondt@anacura.com (I.D.)

**Keywords:** *Caenorhabditis elegans*, lifespan, trehalose, trehalose 6-phosphate synthase, maltose, glucose, glycogen

## Abstract

The *C. elegans* insulin/IGF-1 (insulin-like growth factor 1) signaling mutant *daf-2* recapitulates the dauer metabolic signature—a shift towards lipid and carbohydrate accumulation—which may be linked to its longevity and stress resistance phenotypes. Trehalose, a disaccharide of glucose, is highly upregulated in *daf‑2* mutants and it has been linked to proteome stabilization and protection against heat, cold, desiccation, and hypoxia. Earlier studies suggested that elevated trehalose levels can explain up to 43% of the lifespan extension observed in *daf-2* mutants. Here we demonstrate that trehalose accumulation is responsible for increased osmotolerance, and to some degree thermotolerance, rather than longevity in *daf-2* mutants. This indicates that particular stress resistance phenotypes can be uncoupled from longevity.

## 1. Introduction

The insulin/IGF-1-like signaling (IIS) mutant *daf-2* of the nematode *Caenorhabditis elegans* lives about twice as long as wild type [1]. In this mutant, the reduction of IIS results in nuclear translocation of the FOXO transcription factor DAF-16 [2,3] and subsequent activation of a broad life maintenance program [4]. In nature, this program is also activated during dauer survival under harsh environmental conditions [5] and results in a set of specific phenotypes: A metabolic shift towards carbon storage [6,7,8], increased proteostasis [9], extended lifespan, and increased resistance to a wide variety of biotic and abiotic stressors [10,11,12,13,14,15,16]. It is not entirely clear to what degree these phenotypes are interdependent or intertwined. Maintenance of proteostasis is key to stress resistance and longevity [17]. Hence, increased stress resistance and lifespan are often interlinked [18], although some studies uncoupled both phenotypes [19].

A prominent metabolic characteristic of IIS mutants is their high trehalose level [11,20,21,22,23] and concordant upregulation of trehalose synthesis genes [4,8,24,25]. Trehalose is a natural disaccharide comprised of two α,α-1,1-glucoside bound α-glucose units. It can be found in a wide variety of organisms such as bacteria, fungi, Ecdysozoa, and plants [26]. This disaccharide protects the organism against stress conditions such as heat, cold, desiccation, and hypoxia [22,26,27,28,29,30,31], most likely by acting as a chemical chaperone stabilizing proteins and protecting lipid membranes [27,32,33].

Taken together, trehalose may link many phenotypes of the *C. elegans* life maintenance program: A metabolic shift allows accumulation of this chemical chaperone to increase proteostasis, which in turn promotes both longevity and stress resistance. Several observations support this view. Firstly, in vitro experiments showed that addition of trehalose to wild-type *C. elegans* homogenates reduces protein precipitation by trichloroacetic acid (TCA), which is indicative of its protein stabilization properties. Proteins of *daf‑2* mutants have increased resistance to TCA precipitation compared to wild type and exogenous trehalose addition does not further improve this resistance. This suggests that the high trehalose levels in *daf-2* mutants are responsible for increased protein stability. Indeed, RNAi knockdown of the trehalose synthesis genes *tps-1* and *tps-2* decreases the amount of stabilized proteins significantly [34]. Second, in vivo studies indicated that trehalose supplementation to wild-type worms is capable to extend their lifespan and healthspan. Similarly to the in vitro studies, *daf-2* lifespan is not extended further by trehalose treatment, suggesting that the protein stabilizing property of trehalose may be responsible for *daf-2* longevity. RNAi knockdown of *tps-1* and *tps-2* eliminates about 43% of the lifespan extension in *daf-2* whereas in wild type there is only a minor effect [22]. Third, trehalose has been causally linked to increased stress resistance: Trehalose administration to wild-type worms increases their thermotolerance and the intrinsic thermotolerance of *daf-2* mutants is partially decreased upon knockdown of trehalose synthesis genes [22]. Furthermore, this disaccharide is also responsible for increased osmotolerance and desiccation resistance. The increased osmotolerance observed in the IIS mutant *age-1* is obliterated when trehalose synthesis is knocked down [11]. In addition, the desiccation resistance of *C. elegans* dauer larvae is dependent on trehalose synthesis as *tps-1*;*tps-2* double mutants are not able to successfully undergo anhydrobiosis [27].

Building on the earlier findings that trehalose is a crucial metabolite for *daf-2* longevity and stress resistance, we ask whether specific tissues are crucial for trehalose-dependent longevity and we analyze possible compensatory regulation of other carbohydrates in trehalose synthesis mutants. Finally, the role of trehalose in the resistance to several abiotic stresses is reevaluated.

## 2. Results

### 2.1. Expression Pattern of Trehalose Phosphate Synthases

**Figure 1:**
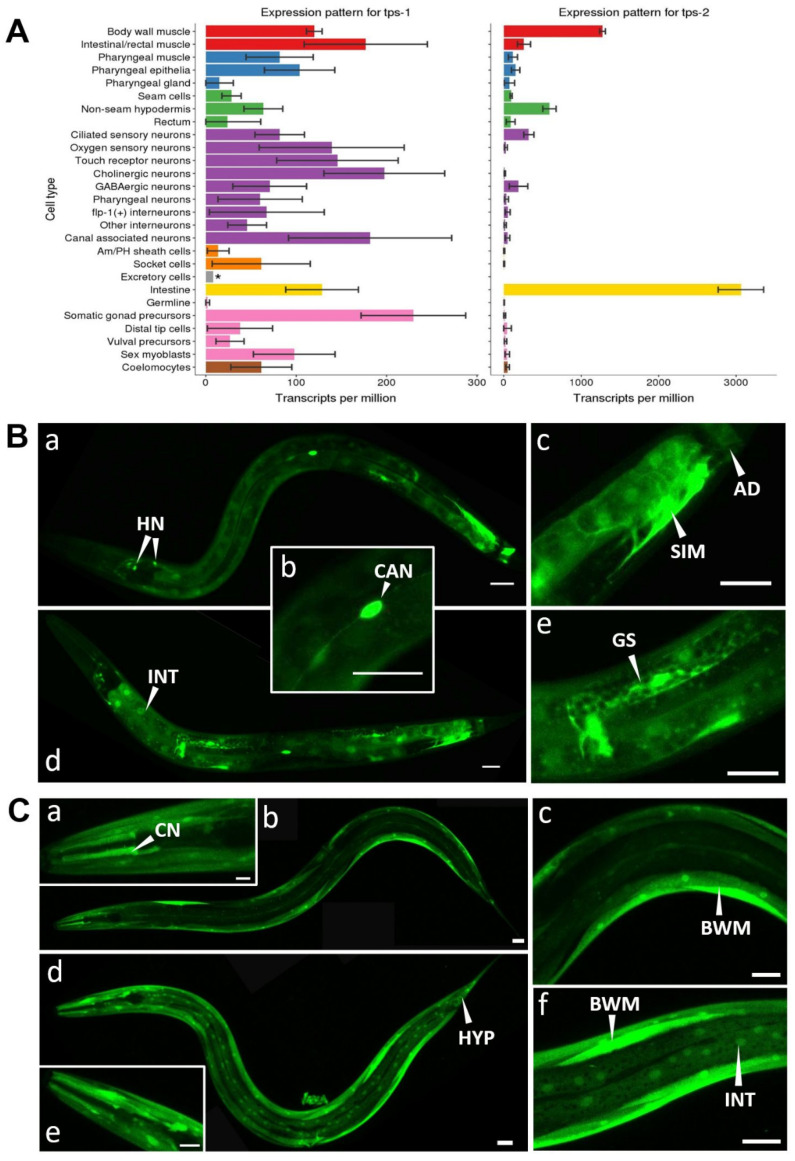
Expression patterns for *tps-1* and *tps-2*: (**A**) *tps* expression based on transcriptional profiling data from [35]. Note that scales of *tps-1* and *tps-2* bar graphs differ tenfold. (**B**) Transcriptional *tps-1p::gfp* reporter strain fed with *E. coli* HT115 expressing empty vector (**a**–**c**) and *daf-2* RNAi (**d**,**e**). (**C**) Transcriptional *tps-2p::gfp* reporter strain fed with *E. coli* HT115 expressing empty vector (**a**–**c**) and *daf-2* RNAi (**d**–**f**). HN, head neuron; CAN, Canal-associated neuron; SIM, stomatointestinal muscle; AD, anal depressor muscle; INT, intestine; GS, gonadal sheath; CN, ciliated neuron; BWM, body wall muscle; HYP, hypodermis. Scale bars are 25 µm.

Trehalose is synthesized by the transfer of a UDP-glucose to glucose-6-phosphate by trehalose phosphate synthase. The resulting trehalose-6-phosphate is converted into trehalose by trehalose phosphatase. *C. elegans* has two trehalose phosphate synthase genes: *tps-1* and *tps-2* and a single trehalose phosphatase, *gob-1*. Based on transcriptional profiling data [35], both *tps* genes likely serve specific tissues in the worm: *tps-1* seems to be the isoform, which is moderately expressed in most tissues, while *tps-2* is highly expressed specifically in the intestine, muscles, and to some extent in the hypodermis and ciliated sensory neurons (Figure 1A). We sought independent confirmation of this pattern by using transcriptional GFP reporters (Figure 1B,C). We observed *tps-1p::gfp* expression in most tissues, including neurons (with likely very high expression in the CAN neurons), somatic gonad, stomatointestinal, and anal depressor muscles, and weak expression in the intestine (Figure 1B). A translational *tps-1::gfp* reporter strain shows a similar pattern but also contains expression in the body wall muscle cells and spermatheca (Figure S1). The *tps-1* expression pattern in adult worms is somewhat different to that reported for dauers, where *tps-1::gfp* was expressed in the hypodermal syncytium, the isthmus of the pharynx, several head neurons, but not in the intestine [36]. *tps-2p::gfp* is strongly expressed in body wall muscles and likely the ciliated sensory neurons, but in contrast to the larval transcriptomics data [35], expression in the intestine and hypodermis is very weak (Figure 1C).

As *tps* expression is known to be strongly upregulated in IIS mutants [4,8,11,22,24,25], it is likely under DAF-16 control. Hence, we analyzed whether RNAi knockdown of the IIS receptor *daf-2* has any effect on *tps* expression level and whether this effect is tissue-specific. The *tps-1p::gfp* expression pattern in *daf-2* RNAi-treated worms is similar to that of the empty-vector control. Expression in the intestine seems somewhat upregulated (Figure 1B and Figure S1) but variation occurs among individuals. Knockdown of *daf-2* does not seem to alter *tps-2p::gfp* expression much (Figure 1C). Hence, we expect that trehalose synthesis in the intestine can be triggered by IIS-dependent activation of *tps-1*, while in other tissues constitutive synthesis may occur.

Interestingly, a similar *tps* expression pattern occurs in starved worms, with variable upregulation of *tps-1* in the intestine and no clear effect on *tps-2* expression [37]. Both *daf‑2* RNAi [38] and starvation [39] result in DAF-16 nuclear localization, explaining the similarity of these patterns.

Finally, we found no evidence of a negative feedback loop between DAF-16 activation and *tps* expression. In wild type worms, DAF-16 is localized in the cytosol while in *daf-2(e1370)*, it accumulates in the nuclei, irrespective of *tps* knockdown (Figure S2).

### 2.2. Longevity of daf-2 Mutants Is Independent of tps Activity

As *daf-2* longevity has been reported to be dependent on trehalose synthesis [22] which occurs in distinct tissues under control of two *tps* isoforms, we wondered whether this lifespan phenotype can be attributed to one single tissue or one *tps* isoform. To this end, we knocked down the two *tps* genes separately as well as combined in different tissues using tissue-specific RNAi strains in both wild type and *daf-2(e1370)* backgrounds. As a positive control, we used systemic RNAi knockdown of *tps* genes, which was expected to lead to a considerable lifespan shortening in *daf-2* mutants while having a negligible effect on wild type lifespan, as reported earlier [22].

**Figure 2:**
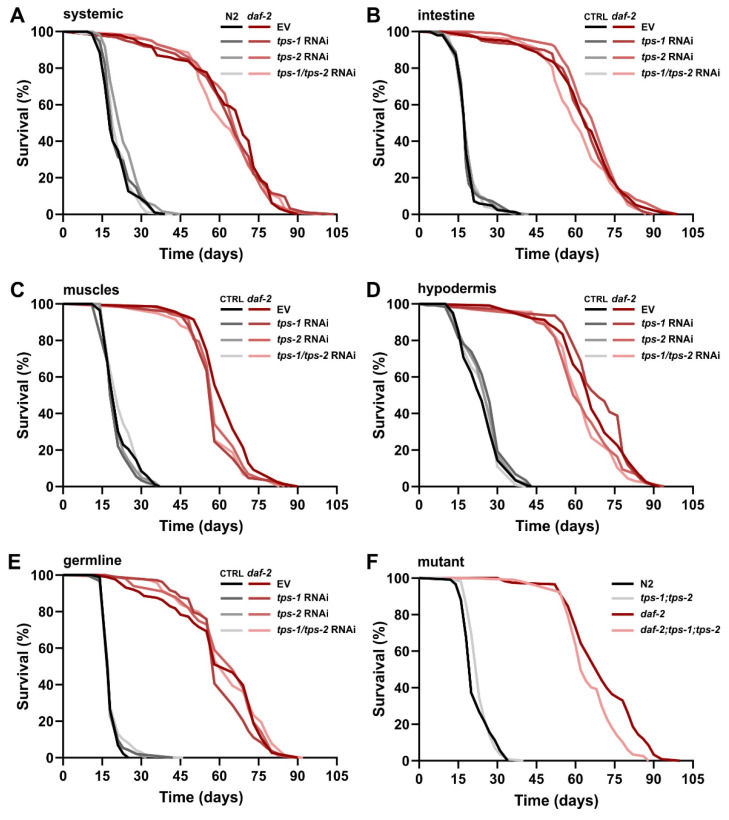
The effect of trehalose synthesis capacity on *daf-2(e1370)* longevity: (**A**–**E**) Tissue-specific RNAi of *tps* genes, (**A**) systemic RNAi, (**B**) intestine-specific RNAi, (**C**) muscle-specific RNAi, (**D**) hypodermis-specific RNAi, (**E**) germline-specific RNAi. Tissue-specific RNAi strains used are listed in Table S1. (**F**) Lifespan of the double *tps* deletion mutant *tps-1(ok373)*;*tps-2(ok526)* in a wild-type and *daf-2(e1370)* background. All data are summarized in Table S2.

Surprisingly, systemic RNAi of both *tps* genes failed to decrease the lifespan of *daf‑2* mutants (Figure 2A). In agreement with this finding, *tps-1* and *tps-2* knockdown—together or separately—in any of the tissues tested (intestine, muscle, hypodermis, germline) did not affect *daf-2* longevity (Figure 2B–E). As the lack of the expected lifespan phenotype may have been caused by low RNAi efficiency, we decided to confirm our experimental results using *tps* mutants (Figure 2F). Deletions in both *tps* genes barely affect *daf-2* longevity: *daf-2* lifespan extension is reduced by only 9% (*p* = 0.00004). In summary, our multiple lifespan experiments (Figure 2A–F) indicate that enhanced trehalose synthesis is not essential for lifespan extension of IIS mutants.

### 2.3. tps-1/2 RNAi in daf-2 Mutants Reduces Trehalose to Wild-Type Levels

As our lifespan experiments are at odds with data published earlier [22] we tested the efficiency of the *tps* deletions and knockdowns by quantifying endogenous trehalose in the worms. We also verified whether trehalose levels are increased in the *daf-2* mutant.

**Figure 3:**
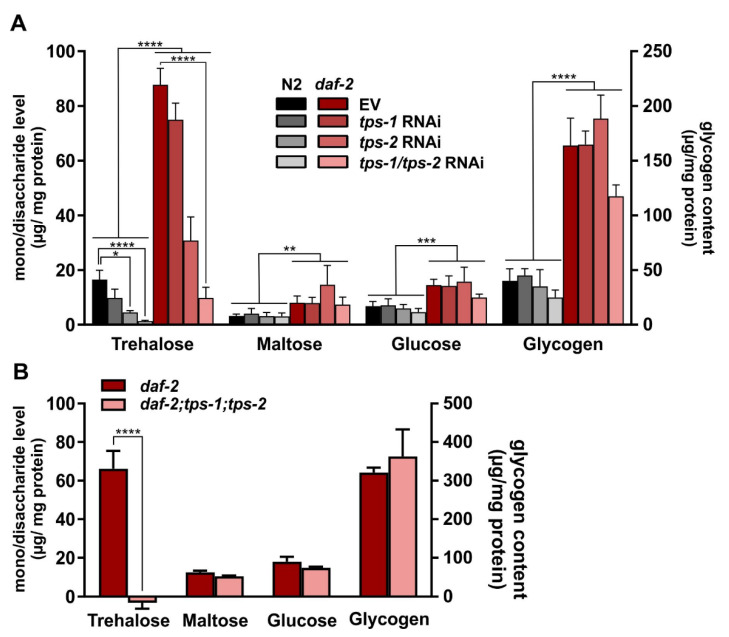
The role of *tps-1* and *tps-2* in worm carbohydrate levels: (**A**) *tps-1* and/or *tps-2* RNAi in wild-type N2 and *daf-2(e1370)* mutants—EV is empty vector control; (**B**) carbohydrates levels in OP50-fed *daf-2(e1370)* and *daf-2(e1370);tps-1(ok373);tps-2(ok526)*. All error bars indicate SEM of three independent replicates. * *p* ≤ 0.05, ** *p* < 0.01, *** *p* < 0.001, **** *p* < 0.0001. Data of all replicates are summarized in Table S3.

Using standard biochemical assays, we found that trehalose levels in *daf-2* mutants are five-fold higher than in wild type (*p* < 0.0001) (Figure 3A), which is a stronger phenotype than the 1.4 to 2.5-fold increases reported before in *daf-2(e1370)* [21,22,23] and the two-fold increase observed in the IIS mutant *age-1* [11]. Knocking down *tps-1* slightly decreases trehalose levels in both N2 and *daf-2*, but this effect does not reach statistical significance. In contrast, *tps-2* inactivation greatly reduces trehalose levels in both strains (*p* ≤ 0.05). This observation is in accordance with the higher expression level of *tps-2* compared to *tps-1* (Figure 1A). Simultaneous knockdown of both *tps* genes yields the strongest decrease in trehalose levels in both strains: A 92% and 89% reduction in wild type and *daf-2*, respectively (*p* < 0.0001). As an additional control, trehalose levels were also determined in the *daf-2;tps-1;tps-2* triple mutant, that seems incapable to produce any trehalose at all (Figure 3B).

Hence, taking together our lifespan analyses and trehalose quantification, we can conclude that increased trehalose levels in *daf-2* mutants are not required to support their longevity.

### 2.4. Maltose, Glucose, and Glycogen Do Not Compensate for Trehalose Reduction

Since knockdown of both *tps* genes resulted in a drastic decrease of worm trehalose, we wondered whether other carbohydrates compensate for this loss and may influence worm physiology and survival. In nematodes, carbohydrate stores primarily occur as glycogen deposits, taking up to 3.3% of the dry body mass [40], but also substantial levels of the monosaccharide glucose are found [41]. Glycogen and glucose are in close proximity to trehalose in the worm’s metabolic network [42]. Hence, we decided to follow the response of these carbohydrates to *tps* knockdown. In our analysis, we also included the other glucose homodimer maltose.

Maltose, glucose, and glycogen levels are not significantly affected by *tps* RNAi, indicating a lack of any clear compensatory reaction (Figure 3A). However, the levels of these three carbohydrates were significantly increased in *daf-2* mutants compared to the wild-type control. Glycogen levels were increased four-fold (*p* < 0.0001), which again is much higher than was previously reported [23,43] although strong increases have been reported in a study using histochemical staining, but were not quantified [7]. Maltose and glucose levels of *daf-2* animals are also significantly elevated compared to those of wild type (*p* = 0.0014 and 0.0003 respectively) indicating a general increase in carbohydrate content in the long-lived mutant (Figure 3A). Furthermore, in the *tps* deletion mutant we find no compensatory carbohydrate response (Figure 3B).

Taken together, reduction in trehalose due to *tps* gene inactivity is not compensated by a substantial increase in the level of any other abundant carbohydrate.

### 2.5. Trehalose Is Required for Increased Osmotic and Heat Stress Resistance in daf-2 Mutants

As the high trehalose level in *daf-2* mutants does not support their longevity, we wondered whether it has any effect on other traits. Besides being long-lived, *daf-2* mutants are known to be resistant to multiple stressors, phenocopying a typical dauer characteristic. Trehalose was shown to be required for heat stress resistance in the *daf-2* mutant [22], for osmotic stress resistance in *age-1*, another IIS mutant [11], and for dauer desiccation survival [27]. Considering the unexpected absence of any effect of trehalose depletion on *daf-2* longevity, we retested the role of trehalose in acute heat stress and osmotic stress survival. We also included an oxidative stress resistance assay as this trait is often linked to longevity and may indicate whether trehalose confers a broad or more specific stress resistance.

**Figure 4:**
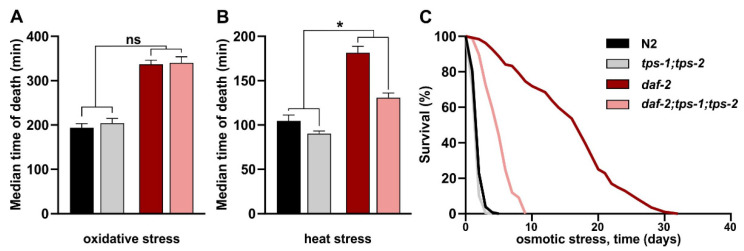
The role of double *tps* deletion *tps-1(ok373);tps-2(ok526)* in stress resistance of wild-type N2 and *daf-2(e1370)*: Stress survival measured with the LFASS method [44] (**A**) oxidative stress, 0.28% *tert*-butyl hydroperoxide (TBHP); (**B**) heat stress, 40 °C; (**C**) osmotic stress survival 500 mM NaCl. Error bars in (**A**,**B**) indicate SEM of three independent replicates. ns: *p* > 0.05, * *p* ≤ 0.05. Survival data of all replicates are summarized in Table S4.

We confirmed that *daf-2* mutants are hyperresistant to all tested stressors (Figure 4A–C, Table S4). *daf-2* resistance to oxidative stress remains unaffected upon *tps* knockout, suggesting that trehalose is not required for the enhanced oxidative stress resistance in *daf-2* (Figure 4A). In contrast, acute heat stress resistance in *daf-2* mutants is partially dependent on trehalose synthesis (Figure 4B), confirming earlier findings [22]. In *daf-2*, the reduction in thermotolerance due to *tps* knockout is about 10% larger than that in wild-type N2 (*p* = 0.0136). Finally, we find that the extremely high resistance to osmotic stress in *daf-2* is largely trehalose-dependent: 77% of this resistance disappears in the double *tps* mutant that is incapable of producing any trehalose (Figure 4C). As wild-type worms also contain some trehalose (Figure 3A), one may expect that wild types should be slightly more resistant to heat and osmotic stress than the *tps-1;tps-2* double mutants. We observed a small but non-significant difference between these samples (*p* = 0.2180 and *p* = 0.9741, for heat and osmotic stress, respectively), likely because trehalose concentrations in wild types are too low to act as an efficient colligative protectant against these stressors.

In summary, we found that high trehalose levels in *daf-2* mutants specifically support their hyper-resistance to osmotic stress, and to some extent heat stress resistance, but have no role in oxidative stress resistance and longevity.

## 3. Discussion

*C. elegans* is a nematode specialized in colonizing spatially limited ephemeral food patches of rotting plant material. In these patches, self-fertilizing hermaphrodites produce rapidly expanding clonal populations that may quickly exhaust the available food [45]. As a response to overcrowding and food deprivation, young larvae enter the dauer stage, a diapause stage specialized in long-term survival and characterized by cessation of feeding and defecation, increased stress resistance, anabolic arrest, decrease in energy consumption, and a metabolic shift towards lipid and carbohydrate storage [46]. These dauers may be transported by invertebrate carriers to a new food patch and hence act as propagules for a new clonal population [47]. The ability of dauers to overcome longer periods of drought by undergoing anhydrobiosis may dramatically increase their success as propagules. Dauer anhydrobiotic survival depends on the synthesis of the disaccharide trehalose, that likely acts as a chemical chaperone to protect membranes and proteins during dehydration [27].

Dauer formation is dependent on Ins/IGF1-like signaling and the IIS mutant *daf‑2(e1370)* shows many dauer-like features during its adult life, including increased stress resistance, long lifespan, and a shift towards lipid and carbohydrate accumulation, including high trehalose levels [8,25].

In this study, we found that trehalose is not required for *daf-2* longevity but it largely contributes to osmotic stress resistance and to some extent to heat stress resistance in these mutants. The independence of *daf-2* longevity to trehalose is at odds with an earlier report [22]. However, in a recent study, RNAi knockdown of *tps-1* and *tps-2* only lead to an 11% reduction of *daf-2* lifespan suggesting a minor contribution of trehalose to IIS longevity [23]. Discrepancies in lifespan data between labs often originate from slightly differing experimental conditions. Although in both the original Honda et al. [22] and our studies, the same *daf-2* allele, same food, and same culturing medium was used, there were a few minor experimental differences (summarized in Table S5). While Honda et al. raised all worms at 20 °C, we cultured young juveniles at 16 °C to avoid dauer formation in the temperature sensitive *daf-2(e1370)* allele. L4 larvae were switched to 20 °C until the end of the experiment. Hence, during adult life, worms of both studies experienced the same temperature, which is reflected in the very similar lifespans obtained by both labs for the N2 as well as *daf-2* strains. Another difference in the protocol is the FUDR concentration used to prevent progeny: Honda et al., used 40 µM while we applied 100 µM. This factor may also not explain the different outcome as in the Seo et al. study [23], 200 µM FUDR was applied and an intermediate lifespan effect of *tps* knockdown was found (Table S5). Thus, there seems to be no correlation between FUDR concentration used and the magnitude of the lifespan effect. Finally, Honda et al. transferred worms to new plates every three days, while in our study, this was done weekly. Given that worms lacking trehalose are more sensitive to starvation effects [37] and osmotic stress (this study), one would expect that *daf-2;tps-1;tps-2* worms would be shorter-lived in our assays, where worms were left on plates longer and thus had increased risk of starvation or drying out. This is clearly not the case, thus transferring frequency is likely not an issue. Taking into consideration the few experimental differences between our and Honda’s study, we have, at this point, no explanation for the contrasting results.

Although *daf-2* mutants are hyper resistant to a wide range of stressors, the high trehalose levels seem to protect specifically against osmotic stress (and to some extent heat stress) while having no effect against oxidative stress. It is likely that the elaborate DAF-16-dependent life maintenance program [4,25] functions in non-overlapping or partially overlapping modules that each enable the worm to cope with a specific challenge from the unfavorable environment. Despite the close correlation between lifespan extension and enhanced stress resistance [18], these phenotypes are often separable [19,48], as evidenced by our study. Finally, of note, despite the fact that trehalose has been linked repeatedly to heat and osmotic stress resistance and a direct mechanistic connection is highly likely—we cannot exclude the possibility that the trehalose synthesizing enzymes *tps-1* and *tps-2* act in an indirect way to promote heat and osmotic stress resistance in *daf-2* mutants. One such possible mechanism is that, as was found in yeast [49], mutation in *tps* results in lowered expression of heat shock proteins upon heat treatment.

The *C. elegans* genome codes for two trehalose phosphate synthase isoforms, *tps‑1* and *tps-2*, showing expression in partially overlapping tissues. Both *tps* isoforms seem under DAF-16 control and upregulated in *daf-2* mutants as repeatedly shown in transcriptomics [4,25,50,51,52,53] and proteomics studies [8,9]. The reason that trehalose synthesis in *C. elegans* is supported by two separate *tps* isoforms under control of the same transcription factor is currently unclear. GFP reporter analysis shows *tps-1* expression in neurons, somatic gonad, intestine, intestine-associated muscles, and body wall muscles. Like *tps-1*, *tps-2* expression was found in the intestine and body wall muscles, but also in the hypodermis and ciliated neurons. In the intestine, hypodermis and body wall muscle, TPS may readily convert the abundant glycogen and triglycerides into trehalose [8,42]. Trehalose may be the main transport sugar in *C. elegans* [25] and is likely distributed to other tissues by facilitated transport via FGT-1, a glucose transporter that is also able to carry trehalose over the plasma membrane [54]. This transporter is expressed in several tissues [55] including the basolateral membrane of the intestine [56], where it could facilitate trehalose release from the intestinal cells into the pseudocoelomic cavity for redistribution of energy throughout the body.

We found strong expression of *tps-1* in the gonadal sheath cells, but not at all in the oocytes and eggs although the latter were reported to contain high levels of trehalose [57]. This suggests maternal provision of trehalose, synthesized in the gonadal sheath, to the germline or oocytes. In the embryo, a non-feeding stage, trehalose may serve as an energy source supporting development. However, as *tps* double mutants are still fertile, alternative energy sources likely exist. Alternatively, the protective properties of trehalose may increase chances of egg survival in an unpredictably fluctuating environment. The maternal provision of substances to the offspring is somewhat reminiscent to the fate of vitellogenin, which is synthesized in the intestine and accumulated in the oocytes [58] and supports L1 diapause survival, not embryo development [59].

In conclusion, we hypothesized that the metabolic switch that occurs in *C. elegans daf-2* mutants, which is typical to microorganisms living in fluctuating environments [60], may be responsible for its extended lifespan [42]. Our experimental results showed that one of the major hallmarks of this metabolic switch, the accumulation of trehalose, is not required for *daf-2* longevity but specifically serves its resistance to osmotic stress and partially supports its thermotolerance. Hence, we show that stress resistance and longevity phenotypes can be uncoupled.

## 4. Materials and Methods

### 4.1. C. elegans Strains and Culture Conditions

*C. elegans* strains were maintained at 16 °C on nutrient agar (Oxoid, CM0003) plates seeded with the *Escherichia coli* strain K12. Strains used in this study are summarized in Table S1. The hypodermis- and germline-sensitive strains in the *daf-2(e1370ts)III* background were obtained by standard crossing methods. Presence of the mutations was verified by PCR analysis and sequencing.

### 4.2. RNAi Assay

RNAi assays were performed using the bacterial feeding assay. Bacterial strains, expressing dsRNA against either *tps-1*, *tps-2* (Ahringer genomic RNAi library), and *daf‑2* (Vidal ORF-RNAi *C. elegans* RNAi library) were grown as previously described [61] with some modifications. Briefly, an RNAi colony was grown at 37 °C in LB medium containing 500 µg/mL carbenicillin (Fisher BioReagents^TM^) for no longer than 18 h. Next, 100 µL of bacteria was seeded on nematode growth medium (NGM) agar plates containing Agar N°1 (2.5% *w*/*v*, Oxoid Limited, Basingstoke, UK), Pepton N-Z-Soy(R) BL4 (0.25% *w*/*v*, Sigma Aldrich, St.-Louis, MO, USA), NaCl (0.3% *w*/*v*), cholesterol (0.0005% *v*/*v*), CaCl_2_ (1 mM), MgSO_4_ (1 mM), K_2_HPO_4_/KH_2_PO_4_ (25 mM, pH 6.0). The NGM plates were supplemented with 25 µg/mL carbenicillin and 1 mM isopropyl-β-D-thiogalactopyranoside (IPTG, Dioxane-free, 1 mM, Fisher BioReagents™, Waltham, MA, USA) final concentration. dsRNA was induced overnight at room temperature.

### 4.3. Microscopy

Worms were picked into 5 µL of a 10-mM levamisol solution on microscope slides with 2% agarose pads. They were immediately covered with a coverslip, sealed with nail polish, and imaged using a Nikon TiE-C2 confocal microscope. The *tps* expression patterns were imaged with a 40x CFI Plan Apochromat (NA 1.25, water immersion) objective and the NIS Elements Imaging software (version 4.30.02). GFP was excited at 488 nm and the emission was collected with a 525-nm band pass filter. A z-series of images was taken (Z-step of 0.75 µm) using consistent imaging settings and a maximum intensity Z-projection was applied using the program Fiji [62]. The resulting Z-projections were stitched using Corel Photo-Paint^TM^ X5 (Version 15.0.0.486). Image tone was adjusted consistently for all images using the contrast enhancement tool (input value clipping 0-100). Compilation of images into panels was conducted in Microsoft PowerPoint.

### 4.4. Lifespan Assay

Worms were synchronized by treating gravid hermaphrodites with alkaline hypochlorite solution [63]. The eggs obtained were shaken (120 rpm) at 16 °C overnight in S‑basal (0.1 M NaCl and 0.05 M potassium phosphate buffer, pH 6) to allow hatching. The next day, L1 animals were transferred to plates with dsRNA-expressing HT115 bacteria (or OP50 for non-RNAi experiments) and allowed to reach L4 stage at 16 °C. This low temperature was chosen to avoid dauer formation in the temperature-sensitive *daf‑2(e1370)* mutant. At L4 stage, the temperature was switched to 20 °C and 100 μM (final concentration) 5-fluorodeoxyuridine (FUDR, Acros Organics, Fair Lawn, NJ, USA) was added to prevent progeny production. The day of L4-to-adult switch was determined as day 0. Worms were transferred to fresh plates on a weekly basis and survival was scored every other day. Worms were scored as dead when they did not respond to gentle prodding with a platinum wire. Animals that crawled off the plates and died were censored. Numbers of scored and censored worms are summarized in Table S2.

### 4.5. Stress Resistance Assays

For all stress assays, worms were grown at 16 °C on NGM plates seeded with *E. coli* OP50 until L4 stage. At L4 stage, the worms were treated with FUDR at a final concentration of 100 µM and transferred to 20 °C until day 2 of adulthood.

#### 4.5.1. Osmotic Stress Assay

To assess the resistance to chronic hyperosmotic stress, day 2 adults were manually transferred onto NGM plates containing 500 mM NaCl [64] and 100 µM FUDR. Survival was scored daily until the most sensitive strains died, after which it was continued every 2–3 days until the end of the experiment. Three independent replicates of approximately 120 worms per strain per replicate were performed.

#### 4.5.2. Heat and Oxidative Stress Assays

Resistance to heat and oxidative stress was assessed by the label-free automated survival scoring (LFASS) approach, which relies on time-lapse measurements of blue death fluorescence (λ_ex_ = 360 nm, λ_em_ = 435 nm) [44]. For both assays, approximately 200 worms were placed per well in a clear-bottom 96-well plate, supplemented with previously frozen OP50 to avoid starvation. Blue fluorescence was measured for each well in 2-min intervals over a time span of 24 h in a Tecan Infinite F Nano^+^ plate reader. For the heat stress assay, the worms were exposed to 40 °C, while in the oxidative stress assay the worms were treated with 0.28% *tert*-butyl hydroperoxide (Luperox^®^ TBH70X, Merck KGaA, Darmstadt, Germany). Median time of death, which corresponds to half-maximal blue fluorescence, was automatically extracted for each well in MATLAB 9.9 (The MathWorks, Inc., Natic, MA, USA), using the LFASS software package developed by Benedetto et al. [44]. GraphPad Prism 9 (GraphPad Software Inc., San Diego, CA, USA) was used for statistical analyses and generating graphs. Both stress assays were run in three independent replicates, each with five technical replicates (wells) per strain.

### 4.6. Carbohydrate Determination in Worm Extracts

Worms were cultured similarly as for survival assays, except for the composition of the NGM. Instead of soy-peptone (Merck KGaA, Darmstadt, Germany) we used bactopeptone (Fisher Scientific, Waltham, MA, USA) to decrease spontaneous dauer formation in the *daf-2* mutants. Day 2 adult hermaphrodites were washed three times in S-basal and once with 2.5 mM EDTA in S-basal to remove bacteria. After washing three more times with distilled water, 100-µL samples were snap frozen in liquid nitrogen. On the day of the analyses, samples were thawed and 100 µL distilled water and approximately 100 mg glass beads (~0.25 mm Ø) were added. Homogenization was performed by bead-beating using a Homogenizer Precellys 24 (Bertin technologies, Montigny-le-Bretonneux, France), shaken at 6800 rpm for 30 s. The debris was pelleted by centrifugation at 14,000 rpm for 2 min at 4 °C. The supernatant was subjected to further analysis. The total amount of soluble protein was determined with a bicinchoninic acid (BCA) assay kit according to the manufacturer’s instructions (Thermo Fisher Scientific, Waltham, MA, USA) and was used for normalization to biomass. The carbohydrate levels were quantified with trehalose and maltose/sucrose/D-glucose assay kits (Megazyme, Bray, Ireland), using a modified protocol. For trehalose assessment, 60 µL of supernatant was heated to 95 °C for 10 min to denature any remaining endogenous enzymes. Next, 60 µL of freshly prepared alkaline borohydride (10 mg/mL in 50 mM sodium hydroxide) was added to reduce the sugars and the sample was incubated at 40 °C for 30 min and shaken at 300 rpm. Next, 150 µL of 200 mM acetic acid was added for neutralization and the pH was buffered with 60 µL of 2 M pH 7.0 imidazole buffer. One hundred and fifty microliters of the final mixture were pipetted into wells of a 96-well microtiter plate (Greiner, Kremsmünster, Austria) in two technical replicates. An identical amount of trehalose standard (0.1809 mg/mL) was included in a separate well. Finally, 70 µL distilled water and 34 µL of assay mix was added to start the reaction. The microtiter plate was loaded to a plate reader (Perkin Elmer Wallac Victor^2^, Waltham, MA, USA) set at 25 °C and the initial (A1) and final (A2) absorbance values (340 nm) were measured in a 60-min interval.

For determination of the maltose and glucose levels, the manufacturer’s protocol for cuvettes was scaled down 10 times for compatibility with 96-well microtiter plates. Endogenous enzyme inactivation was done as described for the trehalose assay. Forty microliters of samples and standards was pipetted into each well and diluted with 170 µL distilled water. To initiate the reaction, 52 µL of assay mix was added. The concentrations of the maltose and glucose standards were 0.19 and 0.4 mg/mL, respectively. The initial (A1) and final (A2) absorbance values were determined at 340 nm with a 60 min-interval.

Glycogen quantification was performed using a commercially available glycogen assay kit (Merck KGaA, Darmstadt, Germany). The samples were homogenized as previously described, but with the addition of 200 µL distilled water instead of 100 µL. After inactivation of endogenous enzymes, homogenates were further diluted five times with distilled water. Ten microliters of this dilution was used for the assay, which was carried out according to the manufacturer’s protocol with minor modifications. Only 1 µL of fluorescent peroxidase substrate was used per sample and the final volume was brought to 50 µL with development buffer. The change in absorbance was measured at 570 nm at 24 °C during 90 min with a 1-min interval. The highest point in the curve was used to determine the amount of glycogen.

All biochemical assays were replicated three times independently.

### 4.7. Data Analysis

For lifespan and osmotic stress resistance experiments, the log-rank (Mantel–Cox) test was performed using the online application for the survival analysis (OASIS2) web-tool as described by Han et al. [65]. Statistical significance of LFASS data was assessed by two-way ANOVA. Post-hoc comparisons were made using Šidák’s multiple comparison test. Graphs were generated using GraphPad Prism 9.0.0 (121). Statistical significance of the carbohydrate data was calculated using two-way ANOVA. For comparisons between treatments and strains Tukey’s and Bonferroni’s multiple comparison tests respectively were applied.

## Data Availability

The data presented in this study are available in the Supplementary Materials, Tables S2–S4.

## Supplementary Materials

The following are available online at https://www.mdpi.com/2218-1989/11/2/105/s1, Figure S1: expression pattern of *tps-1* in a translational *tps-1::egfp* reporter strain, Figure S2: DAF-16::GFP localization in worms with or without *tps* knockdown in wild-type or *daf-2* mutant background, Table S1: strains used in this study, Table S2: mean lifespan of all tested strains, Table S3: carbohydrate analysis, Table S4: stress survival, Table S5: Lifespan experiment setup of three independent studies.
