# Supplementary material for: Elevated Trehalose Levels in C. elegans daf-2 Mutants Increase Stress Resistance, Not Lifespan
Source: Metabolites. 2021 Feb 12;11(2):105. doi: 10.3390/metabo11020105 (PMC7917784; doi:10.3390/metabo11020105)
Supplement: Supplementary file 1 [file metabolites-11-00105-s001.pdf]

Supplementary Material

Elevated Trehalose Levels in *C. elegans daf-2* Mutants Increase Stress Resistance, Not Lifespan

Madina Rasulova †, Aleksandra Zečić †, Jose Manuel Monje Moreno, Lieselot Vandemeulebroucke, Ineke Dhondt and Bart P. Braeckman *

Figure S1.

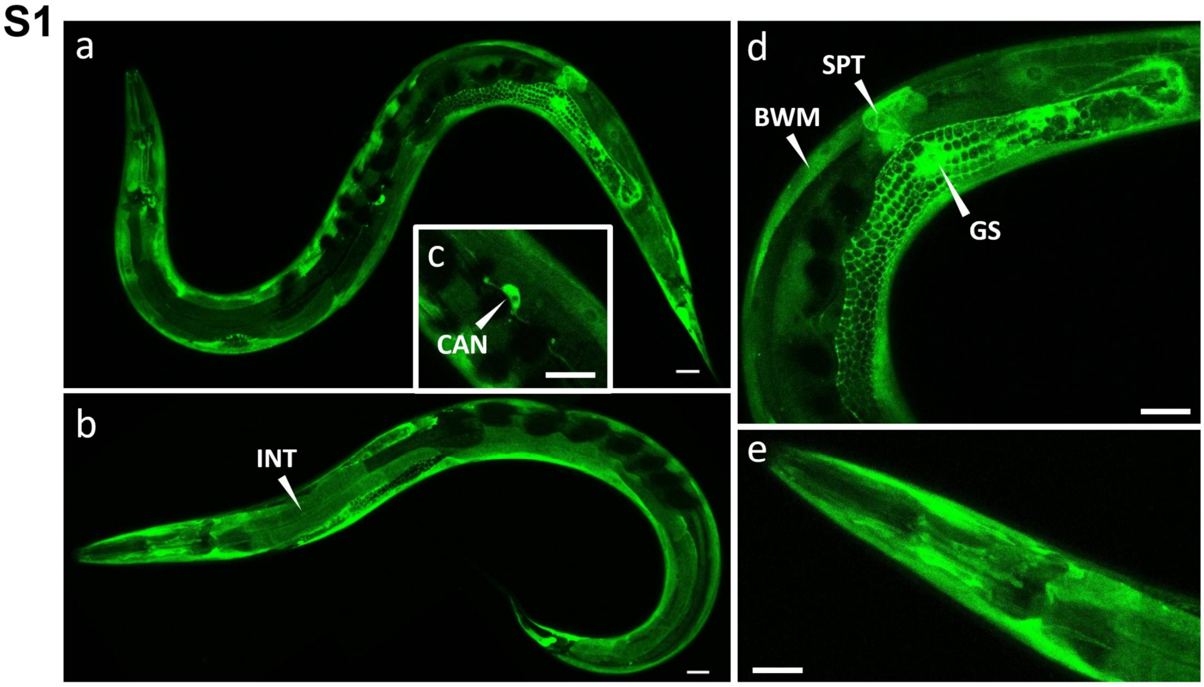

Figure S1. Translational *tps-1::egfp* reporter strain fed with *E. coli* HT115 expressing empty vector (a-c) and *daf-2* RNAi (d,e). CAN, Canal-associated neuron; BWM, body wall muscle; SPT, spermatheca; GS, gonadal sheath; INT, intestine. Scale bars are 25 µm.

Figure S2.

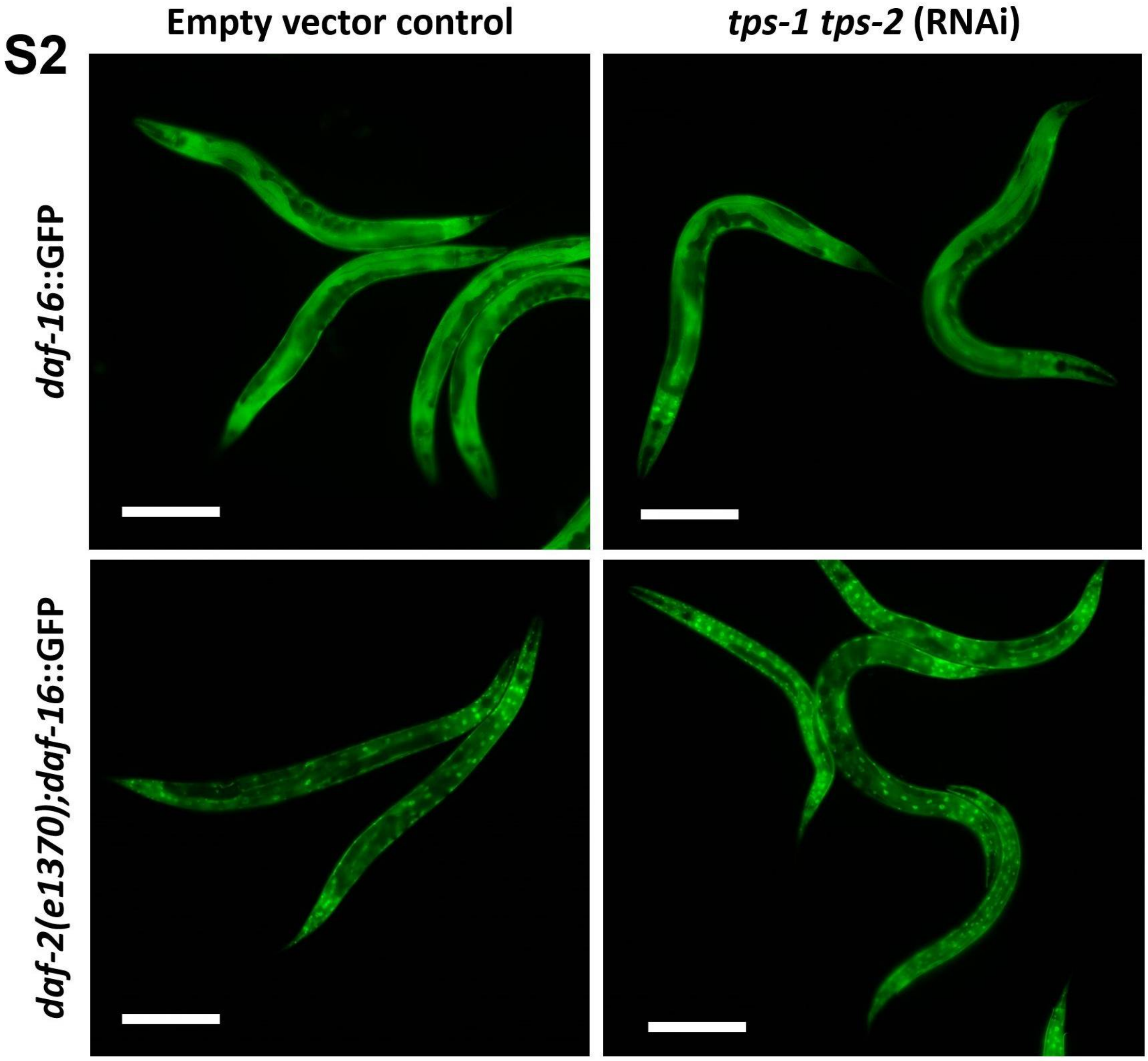

Figure S2. DAF-16::GFP localization in worms with or without *tps* knockdown in wild-type or *daf-2* mutant background. Scale bars are 250 µm.

Table S1. Strains used in this study.

Strain name	Genotype	RNAi-sensitive tissue	Source
N2	wild type	All	Caenorhabditis Genetics Center
CB1370	*daf-2(e1370ts)III*	All	
GR1895	*daf-2(e1370ts)III; mgIs67 [daf-16p::daf-16::GFP + rol-6(su1006)]*	All	
BC14885	*dpy-5(e907)I; sEX14885[rCes tps-1::gfp + pCeh361]*	All	
BC14876	*dpy-5(e907)I; sEX14876[rCes tps-2::gfp + pCeh361]*	All	
NR222	*rde-1(ne219)V; kzIs9[pKK1260(lin-26p::nls::gfp) + pKK1253(lin-26p::rde-1) + pRF6(rol-6(su1006)]*	Hypodermis	
RB798	*rrf-1(ok589)I*	Germline	
TJ356	*zIs356 [daf-16p::daf-16a/b::GFP + rol-6(su1006)]IV*	All	A kind gift from T. Johnson
MGH167	*sid-1(qt9)V;alxls6[vha-6p::sid-1::sl2::gfp]*	Intestine	a kind gift from M. Hansen
MAH410	*daf-2(e1370ts)III;sid-1(qt9)V;alxls6[vha-6p::sid-1::sl2::gfp]*	Intestine	
AGD855	*sid-1(qt9)V;uthls237(myo-3p::tomato+myo-3p::sid-1)*	Muscles	
MAH411	*daf-2(e1370ts)III;sid-1(qt9)V;uthls237(myo-3p::tomato+myo-3p::sid-1)*	Muscles	
JV34	*daf-2(e1370ts)III;rde-1(ne219)V; kzIs9[pKK1260(lin-26p::nls::gfp) + pKK1253(lin-26p::rde-1) + pRF6(rol-6(su1006)]*	Hypodermis	This study
JV31	*rrf-1(ok589)I;daf-2(e1370ts)III*	Germline	
	tps-1p::tps-1::egfp	All	a kind gift from T. Kurzchalia
	daf-2(e1370ts)III; tps-1p::tps-1::egfp	All	
	daf-2(e1370);tps-1(ok373);tps-2(ok526)	All	

Table S2. Mean lifespan of all tested strains.

Mutant/Strain (RNAi active tissue, background)	Treatment (OP50 / RNAi)	Mean lifespan±SEM (days)	Sample size/censored
N2 (systemic)	L4440	20.53±0.61	101/6
	tps-1	21.13±0.62	100/10
	tps-2	23.87 ±0.56	100/4
	tps-1/2	21.11±0.55	126/4
daf-2 (systemic)	L4440	63.92±1.63	100/3
	tps-1	63.34±1.75	101/6
	tps-2	63.86±1.50	100/2
	tps-1/2	62.81±1.56	101/10
MGH167 (intestine, WT)	L4440	18.41±0.50	100/16
	tps-1	18.79±0.57	100/15
	tps-2	18.92±0.53	100/11
	tps-1/2	18.69±0.50	100/13
MAH410 (intestine, *daf-2*)	L4440	63.70±1.55	100/5
	tps-1	63.18±1.44	100/1
	tps-2	67.40±1.23	100/3
	tps-1/2	61.19±1.35	101/4
AGD855 (muscle, WT)	L4440	21.63±0.60	101/16
	tps-1	20.59±0.46	100/10
	tps-2	21.08±0.54	100/17
	tps-1/2	22.51±0.55	102/9
MAH411 (muscle, *daf-2*)	L4440	62.83±1.21	70/2
	tps-1	58.08±1.03	86/1
	tps-2	59.42±1.13	73/0
	tps-1/2	57.64±1.35	59/0
NR222 (hypodermis, WT)	L4440	24.51±0.70	125/7
	tps-1	26.41±0.78	125/6
	tps-2	25.85±0.73	124/14
	tps-1/2	24.88±0.66	124/5
JV34 (hypodermis, *daf-2*)	L4440	66.10±1.19	115/0
	tps-1	68.42±1.33	111/10
	tps-2	62.33±1.49	106/19
	tps-1/2	62.07±1.61	70/3
RB798 (germline, WT)	L4440	18.43±0.22	108/15
	tps-1	18.93±0.44	110/17
	tps-2	18.58±0.32	106/11
	tps-1/2	19.23±0.44	109/9
JV31 (germline, *daf-2*)	L4440	59.90±1.83	101/17
	tps-1	60.34±1.14	112/4
	tps-2	62.35±1.48	100/2
	tps-1/2	62.92±1.45	97/8

N2	OP50	21,82±0,48	123/8
tps-1;tps-2	OP50	23,50±0,39	120/3
daf-2	OP50	70,38±1,19	126/8
daf-2;tps-1;tps-2	OP50	65,79±0,87	122/5

Table S3. Carbohydrate analysis.

Strain	RNAi treatment	Trehalose (µg/mg protein)	Maltose (µg/mg protein)	Glucose (µg/mg protein)	Glycogen (µg/mg protein)
N2	EV	23.18	4.39	10.21	36.44
		11.77	3.40	5.42	23.00
		14.69	1.83	4.90	60.72
	tps-1	16.14	7.69	11.72	49.64
		5.78	3.04	5.76	33.15
		7.45	1.52	3.82	52.67
	tps-2	4.22	5.78	8.91	65.76
		5.71	1.35	4.31	17.48
		3.78	2.39	4.62	21.82
	tps-1/tps-2	0.81	5.53	7.22	38.34
		1.03	2.51	3.78	17.12
		2.05	1.09	2.80	19.52
daf-2	EV	94.14	12.70	18.37	192.47
		93.31	7.24	14.11	184.98
		75.72	4.36	10.95	113.90
	tps-1	77.94	11.95	21.14	156.42
		83.76	5.10	8.53	148.41
		63.15	6.80	12.94	189.13
	tps-2	13.87	28.81	26.15	208.06
		41.99	7.43	8.72	145.87
		36.51	7.67	12.44	211.61
	tps-1/tps-2	2.74	12.90	12.34	121.35
		10.27	5.10	8.91	133.21
		16.32	4.14	8.76	97.55
daf-2	N/A	61.11	12.17	17.25	307.60
		84.23	14.31	22.73	333.78
		53.05	11.17	13.77	
daf-2;tps-1;tps-2	N/A	-0.06	10.41	15.66	432.81
		-9.37	9.88	15.19	292.36
		0.06	11.29	13.56	

Table S4. Stress survival.

Osmotic stress assay (500 mM NaCl)			
**Strain**	**Biological replicate**	**Mean survival ± SE (days)**	**Sample size/censored**
	1	1.92±0.06	124/0
N2	2	2.17±0.08	131/0
	3	2.15±0.06	128/0
	1	1.55±0.05	121/0
tps-1;tps-2	2	2±0.06	124/0
	3	1.91±0.04	136/0
	1	13.06 ±0.64	129/0
daf-2	2	18.89±0.63	124/0
	3	17.29±0.58	139/0
	1	4.78±0.15	132/0
daf-2; tps-1;tps-2	2	5.27±0.2	128/0
	3	5.24±0.17	157/0

Oxidative stress assay (0.28% tBOOH)		
**Strain**	**Biological replicate**	**Median time of death ± SE (min)**
	1	198.02±13.10
N2	2	176.05±5.76
	3	207.36±15.41
	1	215.46±6.63
tps-1;tps-2	2	183.24±6.45
	3	214.12±5.24
	1	339.40±20.38
daf-2	2	319.08±13.74
	3	351.59±22.64
	1	314.12±11.51
daf-2; tps-1;tps-2	2	360.74±5.73
	3	345.31±9.33

Heat stress assay (40°C)		
**Strain**	**Biological replicate**	**Median time of death ± SE (min)**
	1	103.18±2.34
N2	2	94.14±5.09
	3	116.65±5.90
	1	96.3±4.36
tps-1;tps-2	2	87.07±3.44
	3	87.50±4.29
	1	170.69±8.01
daf-2	2	178.29±4.60
	3	195.45±21.69
	1	129.69±5.74
daf-2; tps-1;tps-2	2	122.39±5.66
	3	140.36±5.78

Table S5. Lifespan experiment setup of three independent studies.

	Honda et al. 2010	Seo et al. 2018	This study	
***tps* knockdown**	Systemic RNAi	Systemic RNAi	Systemic RNAi	Mutants
***daf-2* allele**	*e1370*	*e1370*	*e1370*	*e1370*
**Temperature regimen**	20 °C	20 °C	16 °C → 20 °C	16 °C → 20 °C
**Food**	HT115	HT115	HT115	OP50
**Agar**	NGM	NGM	NGM	NGM
**FUDR**	40 µM	200 µM	100 µM	100 µM
**Transfer**	Every 3 days	Not mentioned	Weekly	Weekly
**Lifespan effect of *tps***	**43%** [1]	**11%** [2]	**3%** [1]	**9%** [1]

[1] Calculated as the *tps*-dependent decrease (%) of the *daf-2* lifespan extension.
[2] Calculated as the *tps*-dependent decrease (%) of the total *daf-2* lifespan (parallel N2 data are not available in this study). Using N2 of a non-parallel experiment in this study, we estimated the *tps*-dependent decrease of *daf-2* lifespan extension to be around 20%. While N2 and *daf-2* lifespans in our study and Honda's et al study were very similar, those of the Seo et al., study were much shorter.
